# Infective Endocarditis and Intravenous Drug Users: Never Was and Never Will Be Taken Lightly

**DOI:** 10.7759/cureus.12812

**Published:** 2021-01-20

**Authors:** Tikal Kansara, Monil M Majmundar, Joanna Lenik, Manuel Vista, Shobhana Chaudhari

**Affiliations:** 1 Internal Medicine, Metropolitan Hospital Center, New York Medical College, New York, USA

**Keywords:** infective endocarditis, blood culture negative endocarditis, acute aortic regurgitation

## Abstract

Infective endocarditis (IE) is the infection of the endocardial surface (innermost layer - valves, chordae tendineae, and papillary muscles) of the heart. It usually refers to infection of one or more of the heart valves which may be native or prosthetic. The definition also includes infection on indwelling cardiac devices. Over time, the etiology, as well as causes of IE, have evolved and doubled in numbers because of a greater number of patients with indwelling cardiac devices and central lines. Some characteristic features have remained the same, including intravenous drug users (IVDU) and right-sided IE, fever, or peripheral signs of IE. However, there are instances where the clinical presentation is unique. Here we describe an unusual case of an IVDU patient developing acute decompensated heart failure following acute aortic regurgitation (AR) from IE without fever and right-sided heart or tricuspid valve involvement.

## Introduction

Infective endocarditis (IE) refers to the infection of the endocardium of the heart. Classically, IE is an infection of the native or prosthetic heart valves, but with an increasing number of intracardiac devices and central lines, there is an increase in the incidence of the intracardiac abscess (endocardial abscess) and catheter-related infections. Between 2000 and 2011, the incidence of IE increased from 11 to 15 per 100,000 population in the United States [1] and this trend continues to increase. According to a report by the World Health Organization (WHO), the total number of intravenous drug users (IVDUs) in 2013 was around 12.19 million [2]. These people are at the highest risk for IE.

IE is common in IVDUs. However, there are instances where patients present with atypical characteristics. It is in these patients that the majority of the complications occur. A high degree of suspicion and a low threshold for diagnostic investigations is required to diagnose and rule out IE in IVDUs. Here we describe a case of an IVDU patient with atypical presentation of IE who ultimately required multiple revision surgeries for native aortic valve endocarditis.

## Case presentation

A 33-year-old man, who was in his usual state of health, presented to the emergency department (ED) complaining of shortness of breath. He also complained of chest pain that was pleuritic over the right inframammary and infra-axillary region. In the ensuing days, he had malaise and anorexia without weight loss. One day before the onset of symptoms, the patient admitted he had consumed cocaine through an intravenous route. He had a past medical history of asthma and polysubstance abuse, on a methadone maintenance program.

On arrival to ED, the patient was afebrile (98.8 F), tachycardic, and normotensive. On physical examination, there were bilateral inspiratory crackles over bilateral lung bases. He also had a diastolic murmur over the left lower sternal border. The other systems were noncontributory. Initial tests were notable for a urine drug screen positive for cocaine, marijuana, methadone, and opioids. The serum Troponin T was negative. Blood cultures showed no growth. The liver enzymes were mildly elevated. Complete blood count showed mild hemolytic anemia with hemoglobin of 9.2 g/dL, reticulocytosis (2.93%) with normal white blood cell count. The lactate level was 2.4 mmol/L. The serum lactate dehydrogenase (LDH) was 222U/L, C-reactive protein (CRP) 4.27 mg/dL, erythrocyte sedimentation rate (ESR) 51, and pro-B-type natriuretic peptide (proBNP) 3866 pg/ml. Chest radiograph revealed mixed interstitial and air space disease throughout both lungs. The patient was started on ceftriaxone and azithromycin empirically considering sepsis due to pneumonia.

Further diagnostics were done including an infectious panel with positive results for hepatitis C and *Chlamydia psittaci* IgM antibody titer turning out positive (>1:10). Mycoplasma IgM antibody, influenza polymerase chain reaction (PCR), HIV, hepatitis B, and Quantiferon tuberculosis were negative. Computed tomography scan of the chest with intravenous contrast revealed bilateral effusion, ground-glass opacities, reticulonodular pattern, lymphadenopathy, hepatosplenomegaly. Echocardiography (ECHO) showed severe aortic regurgitation, normal left ventricular ejection fraction (>55%), and right ventricular systolic pressure elevated at >60mm Hg consistent with severe pulmonary hypertension, and 1 cm x 0.5 cm vegetation on the aortic valve (Video 1-4).

**Video 1:** Infective endocarditis at aortic valve seen as thickening of aortic valve

**Video 2:** Infective endocarditis seen as thickening and incomplete closure of aortic valve in this parasternal short axis view

**Video 3:** Aortic regurgitation seen in this long axis view with Doppler which was a consequence of infective endocarditis

**Video 4:** Aortic regurgitation seen in this four chamber Doppler view which is a consequence of infective endocarditis

Given clinical and ECHO finding suggestive for infective endocarditis of aortic valve (AV), the cardiology team recommended cardiothoracic surgery evaluation for AV replacement. Intravenous furosemide was started and the patient was prepared for aortic valve replacement. He underwent a successful mechanical aortic valve replacement. The native valve culture did not grow any organism. 

Follow-up

Upon discharge, the patient had a long and complicated course. Due to his non-adherence to treatment and continued use of intravenous drugs, he later developed recurrent AV endocarditis with multiple organisms. Blood cultures isolated include *Streptococcus sanguinis*, *Candida albicans*, and *Streptococcus mutans*. He underwent multiple aortic valve repairs. On his latest admission, the blood cultures grew *Haemophilus parainfluenzae*, and the patient was treated with six weeks of intravenous antibiotics. Eventually, he developed prosthetic AV stenosis and acute occipital lobe infarct. He is being closely followed by the cardiology team. 

## Discussion

Diagnosis of infective endocarditis is largely based on Duke Criteria, which includes pathologic findings on blood or valve vegetation cultures and clinical findings. Infective endocarditis commonly presents with fever, occurring in up to 96% of cases [3]. It is usually absent on patients started on antibiotics, antipyretics, elderly patients, with heart failure or renal failure. The disease itself had been known to have unusual and non-specific presentations with case reports citing neuropsychiatric manifestations such as symptoms of transient ischemic attack (TIA) or stroke, seizures, or agitation [4].

Clinicians often rely on the presence of fever as a clue regarding a likely infectious process. Hence, in afebrile cases, physicians tend to miss this diagnosis. The patient’s initial presentation with shortness of breath without a history of fever and leukocytosis steered investigation towards new-onset congestive heart failure with fluid overload. The history of cocaine abuse was in favor of the diagnosis. Also, the possible presence of underlying malignancy was investigated. The infectious panel should include mycoplasmas, influenza, hepatitis B, and tuberculosis as the etiology of decompensation. For our patient, only *Chlamydia psittaci* turned out to be positive. A thorough physical examination revealed the presence of a diastolic murmur and prompted an echocardiogram to be done. Endocarditis with negative blood cultures are responsible for 5% of the cases, and the commonly involved organisms include species of *Bartonella*, *Coxiella burnetti*, bacteria of the HACEK group (*Haemophilus*, *Actinobacillus*, *Cardiobacterium*, *Eikenella*, *Kingella*), *Tropheryma whipplei*, species of *Legionella*, species of *Brucella* and fungi [5]. The afebrile state is an interesting observation in this case. So-called "euthermic endocarditis," could be subject to a delay in diagnosis and initiation of appropriate antimicrobial and/or surgical therapy, resulting in an increased risk of IE-related complications and poorer outcomes [6]. This phenomenon is still very much under-investigated, and the pathogenesis needs to be explored. Desimone et al. in 2013 reported an association between afebrile patients and being immunocompromised [6]. In light of this discovery it is critical to remember that although fever is the most common clinical finding in endocarditis, its absence should not mislead the clinician to discard this diagnosis. It is therefore recommended to obtain blood cultures in patients with organic cardiac murmurs who display any unexplained findings [7].

Further, a coordinated approach to addiction services and rehabilitation are required in IVDUs with IE. This approach is largely forgotten and shadowed by other critical and life-saving management and approaches. However, taking our patient as an example, there comes a need to emphasize a coordinated and systematic multidisciplinary approach to prevent complications and new IE in IVDUs. 

## Conclusions

Diagnosis of endocarditis can become challenging in patients with an atypical clinical presentation. Due to the nature of the disease that can have an unusual presentation, clinicians should be aware of the variety of presentations a patient with suspected IE could have as disease prognosis relies on a prompt diagnosis. A patient without fever does not necessarily rule out IE if the clinical suspicion is high.
